# Man and the machine rise to the spike‐wave. Commentary on “An automated, machine learning‐based detection algorithm for spike‐wave discharges (SWDs) in a mouse model of absence epilepsy.”

**DOI:** 10.1002/epi4.12428

**Published:** 2020-08-26

**Authors:** Kevin M. Kelly

**Affiliations:** ^1^ Neuroscience Institute Allegheny Health Network Pittsburgh PA USA

Anyone who has spent a considerable amount of time in visual analysis and manual scoring of EEG events of either human or animal long‐term EEG recordings fully understands that the process is not entirely a labor of love. Manual review of large volumes of EEG data, whether for detection of ictal discharges, epileptiform activity, or abnormal slowing, is notoriously time‐consuming, fatigue‐inducing, and frequently inexact. Numerous analytical software programs have been developed over the last several decades to aid visual review of continuous EEG recordings and have contributed significantly to the precision and accuracy of event detection. However, event detection by even the best algorithms remains an imperfect science. The ongoing need to build better analytical “mousetraps” for EEG event capture continues to be a daunting challenge for epileptologists, computer scientists, and basic researchers.

Such a challenge was embraced wholeheartedly by Pfammatter and colleagues.^1^ They built an automated, machine learning‐based detection algorithm for spike‐wave discharges (SWDs) in the γ2R43Q mouse model, a GABA_A_ receptor knock‐in mutation that generates ~6 Hz SWDs (absence epilepsy) in the animal. As many are aware, SWDs in rodent models of acquired epilepsy continue to be an active topic of discussion and interpretation.^2^ The fundamental uncertainty of this ongoing issue is what is or is not an actual SWD associated with “absence” discharges. The authors of this study recognized the critical importance of rigor in defining SWDs in their model and the associated need to develop robust, automated methods for their detection. They contended that the true definition of SWDs should arise from comparison of rigorously definable events, such as a set of predictor variables, with other known EEG features, such as the sleep‐wake cycle, or treatments that alter SWDs, for example, ethosuximide. Their deliberative efforts resulted in a highly integrated system of confidence‐based scoring of events along a continuum, in addition to a binary classification (SWD/non‐SWD), that reflected physiologically relevant EEG features of normal behavioral states and matched scoring characteristics of human reviewers.

Their efforts began with two of the authors manually scoring 24‐hour unannotated EEG records outside of the EEG training sets for the presence of SWDs—4 from γ2R43Q mice (RQ) and 1 without the mutation but with the same genetic background (RR)—in order to facilitate the development and performance validation of a 2‐stage algorithm. For Stage 1, they constructed a support vector machine (SVM)‐based algorithm, a learning tool used to analyze data and separate groups of events for classification purposes, after being trained with a set of prescribed labels. Importantly, as the authors describe, the boundaries of the classification space are defined by events assigned as support vectors; the location of each event relative to the nearest support vectors can be used for its classification, and the distance from each event to its nearest support vector can be used as a proxy for confidence in its assigned classification. The algorithm first identified 2500 putative SWDs based on frequency‐ and amplitude‐based threshold criteria from 10, 24‐hour EEG records from five animals (3 RQ and 2 RR). From these 2500 presumed SWD events, 2050 unique events were randomly chosen, from which 50 events were selected to be randomly repeated 10 times each. Combining these events together, a new set of 2500 SWD events was presented to four experienced reviewers, including an epileptologist, to score the detections as SWD or non‐SWD. In so doing, intra‐ and interrater scoring consistencies could be calculated.

From the putative events detected by Stage 1, 12 predictor variables were extracted for use in Stage 2 of the algorithm, intended to classify events with an SVM. Using a wavelet transform of each event and targeting the typical frequency of SWDs in mice and several of its harmonics, the 12 predictor variables were calculated from four frequency bands using the mean, standard deviation, and maximum value for each event. Together with the results of the human scoring, the SVM was trained to classify each event as either SWD or non‐SWD and assigned confidence scores to each event.

Event detection in EEG recordings by expert reviewers runs along a spectrum of difficulty depending on the nature of the event. For example, ictal discharges in human EEG recordings that demonstrate “classical” findings such as an abrupt low‐voltage/high‐frequency discharge followed by evolving changes in amplitude and frequency and post‐ictal slowing usually can be recognized without much difficulty. Accurate identification of epileptiform activity, or pathological slowing versus that of variable drowsiness, becomes an entirely different matter. The same challenge is no less true when trying to identify SWDs in animal EEG recordings. Although SWDs are commonly found in both inbred and outbred rodent strains^3^ and many are easily discerned at a glance, others are clearly indeterminate to the human eye as is demonstrated by EEG traces shown by the authors in this study. They hurdled this barrier by use of a wavelet transform of each potential SWD—a method previously applied to rat^4^, ^5^ and mouse SWD models^6^—enabling them to calculate predictor variables that the SVM combined with human scoring to generate a confidence‐based classification result.

Expert review of EEG tracings is a critical component of the training and validation of an algorithm for event detection and is considered a “gold standard” in investigations.^7^ However, this process is not immune to the vagaries of intra‐ and interrater variability.^8^ Not to be underestimated in this study is the authors’ awareness of the importance of assessing human intra‐ and interrater consistencies in the scoring of SWD events and using its strengths and limitations in producing a rigorously defined classification system of SWD/non‐SWD with various degrees of detection confidence. Perhaps the authors rightly recognize that the incorporation of intra‐ and interrater consistencies in this study is its most important contribution to the field of event detection in absence epilepsy.

In summary, Pfammatter and colleagues have produced an exceptional report that contributes substantially not only to the study of rodent SWDs but potentially more broadly to experimental studies of acquired epilepsy and studies focused on EEG event detection in various clinical settings. They are commended for their efforts.

## CONFLICTS OF INTEREST

I have no conflicts to disclose. I confirm that I have read the Journal's position on issues involved in ethical publication and affirm that this report is consistent with those guidelines.

Read the winning article ‐ https://onlinelibrary.wiley.com/doi/abs/10.1002/epi4.12303
